# Ocelot (*Leopardus pardalis*) Density in Central Amazonia

**DOI:** 10.1371/journal.pone.0154624

**Published:** 2016-05-18

**Authors:** Daniel Gomes da Rocha, Rahel Sollmann, Emiliano Esterci Ramalho, Renata Ilha, Cedric K. W. Tan

**Affiliations:** 1 Wildlife Conservation Research Unit, Department of Zoology, University of Oxford, Tubney, United Kingdom; 2 Grupo de Ecologia e Conservação de Felinos na Amazônia, Instituto de Desenvolvimento Sustentável Mamirauá, Tefé, Amazonas, Brazil; 3 Department of Wildlife, Fish and Conservation Biology, University of California Davis, Davis, CA, United States of America; 4 Instituto Pró-Carnívoros, Atibaia, São Paulo, Brazil; 5 Departmento de Ecologia, Instituto Nacional de Pesquisas da Amazônia, Manaus, Amazonas, Brazil; Oregon State University, UNITED STATES

## Abstract

Ocelots (*Leopardus pardalis*) are presumed to be the most abundant of the wild cats throughout their distribution range and to play an important role in the dynamics of sympatric small-felid populations. However, ocelot ecological information is limited, particularly for the Amazon. We conducted three camera-trap surveys during three consecutive dry seasons to estimate ocelot density in Amanã Reserve, Central Amazonia, Brazil. We implemented a spatial capture-recapture (SCR) model that shared detection parameters among surveys. A total effort of 7020 camera-trap days resulted in 93 independent ocelot records. The estimate of ocelot density in Amanã Reserve (24.84 ± SE 6.27 ocelots per 100 km^2^) was lower than at other sites in the Amazon and also lower than that expected from a correlation of density with latitude and rainfall. We also discuss the importance of using common parameters for survey scenarios with low recapture rates. This is the first density estimate for ocelots in the Brazilian Amazon, which is an important stronghold for the species.

## Introduction

The ocelot (*Leopardus pardalis*) is a medium-sized Neotropical spotted cat that is currently distributed from southern Texas (USA), throughout Central America and the Amazon, to northern Argentina. During the 1960s and 1970s, ocelots were heavily hunted for the fur trade, causing drastic declines in abundance across their range [1]. Once considered Vulnerable on the IUCN Red list, the ocelot is now a species of Least Concern [2,3] owing to bans on international fur trade. Today, habitat loss continues to threaten its existence, yet population data on this species is scarce. Even though the Brazilian Amazon constitutes roughly half of the ocelot’s range, ecological information of the species in the region is limited and there are no population estimates of ocelots in this region.

Ocelots are terrestrial, mostly nocturnal and solitary animals that present a high degree of plasticity in terms of habitat, occurring in dense forests, open savannas (Brazilian cerrado), Pantanal, Chaco and even in the semi-arid Caatinga [4–7]. The species is also present in anthropogenically modified habitats such as agricultural landscapes, although in such areas it appears to be strongly associated with dense habitat cover [1,8,9]. Its diet is based on small and medium-sized mammals, varying according to availability [8,10,11]. Ocelot home range sizes vary from 1.8 to 38.8 km^2^, with males usually having larger home ranges than females [8,12,13], and densities range from 3 to more than 90 individuals per 100 km^2^ [14]. Ocelots are believed to be the most abundant of the wild cats throughout the species’ distribution [15,16], and are suspected to play an important regulatory role in the dynamics of sympatric small-felid populations [9] through intra-guild killing [17] and competition [18].

Baseline estimates of population density across a species’ range help in the understanding of broad spatial patterns in a species’ distribution, providing important insights on the species’ habitat/ecological requirements. Additionally, if monitored over time, density estimates can reveal population trends. Information on spatial and temporal patterns on a species density are crucial for management and conservation initiatives [14,19]. However, estimating population densities of solitary, elusive and forest dwelling carnivores such as the ocelot, can be challenging [20,21]. Camera trapping in combination with capture-recapture modelling has been the most successful approach to studying naturally marked felid populations [20,22,23].

In this study, we estimated ocelot density using closed population spatial capture-recapture models (SCR, [24]) based on data from three camera-trap surveys carried out in the Amanã Sustainable Development Reserve, Central Brazilian Amazon. Specifically, we implemented a SCR model that shared detection parameters between surveys. For dataset with low recapture rates, this approach improves density estimation [25,26].

Throughout its range, ocelot density has been shown to be positively correlated with rainfall and negatively correlated with latitude [14]. In comparison with published studies of ocelot populations, our study site in the Amanã Reserve has one of the highest average annual precipitation and lowest latitude. Therefore, we expect the density of ocelots in our study site to be amongst the highest reported densities for the species.

## Methods

### Study area

The camera-trap surveys were conducted in the Amanã Sustainable Development Reserve (2°21’S, 64°16’W) located between the Negro and Amazon Rivers. Amanã Reserve covers 2.350.000 ha of pristine rainforest near the confluence of the Amazon and Japurá Rivers. The surveyed area is composed of a mosaic of non-flooded (*terra firme*) forests and forests that are seasonally flooded by black-water rivers (*Igapó*). Approximately 84% of the reserve is covered by *terra firme* forests. The climate in the region is tropical humid, with an average monthly temperature of 26°C and average annual precipitation of 2373 mm [27]. The Amanã Reserve, together with the Jaú National Park to the east and the Mamirauá Sustainable Development Reserve, forms one of the largest continuous blocks of protected tropical forest in the world and the core of the Amazon Biosphere Reserve.

Entry permission to the Amanã Sustainable Development Reserve was granted by the Instituto de Desenvolvimento Sustentável Mamirauá. This study did not involve biological samples and animal handling, nor did it interfere with the animals’ natural behaviour, thus no ethical approval was required.

### Camera-trap surveys

**Fig 1:**
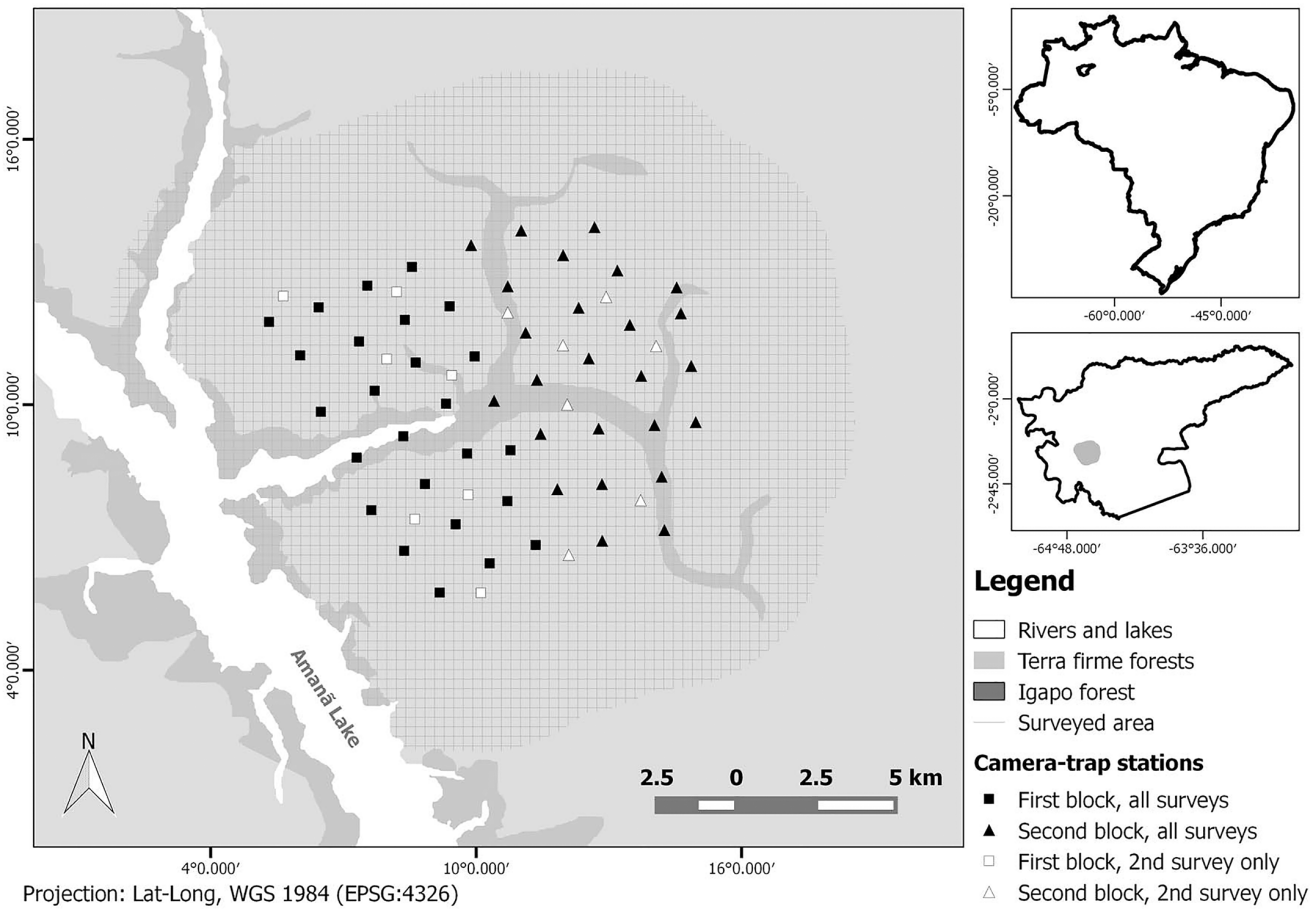
Map of the study area showing the location of camera-trap sites in Amanã Sustainable Development Reserve. White squares and white triangles indicate locations of camera-traps deployed only during the second survey (2014). The insets show the position of Amanã Reserve in Brazil, and the position of our survey area within Amanã Reserve.

We conducted camera-trap surveys on the edges of Amanã Lake during three consecutive dry seasons, when the water level in the region was low. All surveys were originally designed to estimate jaguar density (Fig 1). We carried out the first survey from January to March 2013 (83 days), with a sampling effort of 1909 camera-trap days. The sampling grid had 50 camera-trap stations, covering an area of 130.8 km^2^ (minimum convex polygon). For logistical reasons, we divided the sampling area into two contiguous trapping blocks containing 25 baited camera-trap stations each, with average spacing between stations of 1660.0 m (SD = 226.42 m). Each station consisted of two cameras (model PC800 Hyperfire, Reconyx Inc., Holmen, Wisconsin) facing each other 4–5 m apart. The bait was a mixture of sardine and eggs (~200ml) which was located at the center between the two cameras, inside a container, largely inaccessible for consumption and fixed to the ground.

We carried out the second camera-trap survey from December 2013 to April 2014 (120 days). We set up the same sampling grid as in the previous survey and within that grid we added seven unbaited camera-trap stations in each trapping block, totalling an effort of 2985 camera-traps days. The purpose of those extra unbaited camera-trap stations was to investigate the effect of baits on felid capture rates. By increasing the camera-trap density we reduced the average spacing between stations to 1245.7 m (SD = 262.4 m) and slightly increased the sampling area to 134.9 km^2^ (Fig 1). We used generalised linear models (GLM) with a Poisson distribution to evaluate the effect of the bait on the number of records of ocelots. Our results revealed that there was no significant difference in capture rates between baited and unbaited camera-traps stations (N = 64, slope = 0.44, p = 0.3). Therefore, we included data from these stations in our analyses without accounting for the fact that they were not baited.

For the last survey, we set up the camera-trap stations the same manner as in the first survey (Fig 1) but used no bait, because we considered it ineffective in increasing ocelot records based on data from the previous survey. The cameras were active from January to April 2015 (91 days), totalling a sampling effort of 2126 camera-trap days.

For all surveys, we set cameras to function 24 h per day and take one photo per second when triggered. We visited stations every 14 days to change batteries, download photos and refresh bait. Where possible, we targeted natural animal paths for camera-trap placement, with the exception of three stations that we installed on research trails (5 km long, 2–3 m wide and regularly maintained).

### Estimation of ocelot density

We identified individual ocelots using their unique coat patterns (for details see [4]) to create individual-by-trap encounter histories, containing the number of records of an individual at a given trap. We excluded records that we were unable to precisely identify at individual level.

We analyzed these data using closed population spatially explicit capture-recapture models (SCR). SCR models assume that animals have approximately circular and randomly distributed home ranges [28]. These models use the spatial location of captures to estimate the location of the home range center (or activity center) of each individual. The encounter rate of an individual *i* at a camera trap *j*, *λ*_*ij*_, is assumed to decline as the distance from the home range center increases, following a detection function. A common detection function is the half normal function, which is defined by the baseline encounter rate *λ*_*0*_ (encounter rate at a–hypothetical–camera-trap located at an individual’s activity center) and a movement parameter σ, which is related to the average home range radius (for detailed description of parameters, see [29]). The σ is estimated based on information about animal movement provided by individuals that were captured at more the one camera-trap station.

Because few animals were recaptured within each survey (see Results), we combined data from the three surveys to estimate detection parameters. We specified the model to estimate density separately for each survey, but producing one shared encounter rate (λ_0_) and movement parameter (σ) estimate for all three surveys. The advantage of this approach is that the parameters are estimated based on a larger sample size. By sharing parameters across surveys we assume that home range sizes and detection probabilities are similar across surveys, which is highly plausible because we carried out the surveys during the same period (December-April) of consecutive years, in exactly the same location. Further, we are not aware of any significant biological or environmental change in the area during the study.

We implemented SCR models in a likelihood framework using the R package *secr* version 2.8 [30] in R 3.1.2 [31]. We defined the state space by buffering outermost camera-trap stations by 6300 m. This corresponds to approximately 3 x σ, which should be large enough to contain all potential home range centers of ocelots exposed to our sampling grid [24]. We superimposed a grid mesh of 250 m x 250 m cells over the state space and used a habitat mask to exclude water surface areas. To calculate ocelot trap success, we divided the number of ocelot captures by the total of trap nights times 100.

## Results

In the first survey we obtained 26 independent records of 19 individuals (10 males, 8 females and 1 unknown sex). In the second survey we obtained 45 records of 30 individuals (13 males, 14 females and 3 unknown sex). In the third survey we obtained 22 records of 17 individuals (11 males, 6 females and 2 unknown sex). Five records from the second survey and two from the third survey were discarded due to ambiguous individual identification. We obtained 7 recaptures (of 5 males) in the first survey, 10 recaptures (of 1 male, 4 females and 1 unknown sex) in the second and 3 recaptures (of 3 males) in the third survey. In total, 9 individuals were captured at more than one camera-trap station, 5 males in the first survey, 1 female in the second and 3 males in the third survey. Six individuals were recaptured multiple times at the same station (2 males, 3 females, 1 unknown sex). Overall ocelot trap success was 1.45 ocelot captures per 100 trap nights.

There was no significant difference in density between surveys as 95% CI of density estimates for all surveys largely overlapped (Table 1). The average ocelot density in this study was 24.84 ± SE 6.27 ocelots per 100 km^2^. The ocelot movement parameter *σ* was 2.2 ± SE 0.3 km and estimated baseline encounter rate *λ*_*0*_ was 0.002 ± SE 0.001. Ocelots were the most frequently recorded carnivores in our study.

**Table 1:** Ocelot density estimate with standard error (SE) and 95% confidence interval (Lower and Upper) of parameters for spatial capture recapture model fit to camera trapping data from Amanã Reserve. Data from the three surveys were used to estimate the shared movement parameter σ and encounter rate λ_0._ Density is reported in ocelots per 100 km^2^.

	Estimate	SE	Lower	Upper
shared σ	2.213	0.331	1.652	2.963
shared λ_0_	0.002	0.001	0.001	0.003
Density 2013	25.485	8.467	13.516	48.052
Density 2014	28.182	7.976	16.357	48.558
Density 2015	20.860	7.080	10.921	39.844

For comparison, we provide estimates from a model with independent detection parameters in the supporting information, but we caution that estimates from this independent model are unreliable due to low sample size, particularly for the 2014 survey.

## Discussion

This is the first study to estimate ocelot density in the Central Amazon, which is considered a stronghold for the species [2]. Our results revealed a density estimate of 24.84 ± SE 6.27 ocelots per 100 km^2^. We also showed that when it is possible to assume that parameters remain constant across surveys, sharing parameters can allow density estimation when the data is not sufficient to produce reliable estimates of model parameters for each survey separately, which was the case of the 2014 survey.

Sparse data sets are common in density studies of smaller felids based on trap design originally focused on larger species [25,32], and this is usually due to wide camera-trap spacing. One of the consequences of sparse data is that the movement parameter is poorly estimated, increasing imprecision for SCR models [33]. There was no estimate of ocelot home range size available from our study region to evaluate the suitability of camera-trap spacing for ocelot surveys in the present study. However, our camera-trap spacing was conservative compared to the spacing used by other ocelot density studies that applied spatial capture-recapture models (up to 4 km, [34]). Moreover, in our study the camera-trap spacing was not wider than twice the movement parameter, as recommended by Sollmann et al. [35].

The model used in our study estimated the movement parameter based on a larger sample size, by combining information of movements across surveys. However, most of the movements between camera-trap stations in this study were of males, whereas recaptures at the same station were of females. This is not surprising as male ocelots usually have larger home ranges and move longer distances than females [8,12,13]. Modelling sex-specific movement parameters would be ideal in this case [36], but was not possible due to the low number of spatial recaptures of females. Therefore, our movement parameter might be overestimated (biased towards males). In that case, density in our study would likely be underestimated and should therefore serve as a conservative number [37].

Ocelot density studies using (non-spatial) capture-recapture models report estimates varying from 2.9 individuals per 100 km^2^ in Mexican tropical deciduous forests [32] up to 94.7 individuals per km^2^ in the northern Peruvian Amazon [38]. The only other study that investigated ocelot density in the Amazon region estimated 80 individuals per 100 km^2^ (based on telemetry data, [8]). Our ocelot density estimate based on SCR models in Amanã Reserve was lower compared to previous studies in the Amazon. This lower ocelot density in Amanã Reserve may in part be related to differences in analytical techniques between the studies. Most of those previous studies were based on non-spatial capture-recapture models (Table 2), which have been shown to lead to higher density estimates compared with spatial capture-recapture models [34,39]. This is because the former method fails to account adequately for individual movement. Estimates of ocelot density for the present study using non-spatial capture-recapture models were as high as 40 ocelots per 100 km^2^ (see S1 File for details on non-spatial capture-recapture analyses and results), which are still lower than that expected for sites in the Amazon [14].

**Table 2:** Ocelot density estimate comparison. Study sites where camera-trap surveys in combination with capture-recapture models have been used to estimate ocelot density. Studies are listed chronologically. Density is reported in ocelots per 100 km^2^. Asterisk (*) indicate that the density is the average of density estimates from more than one surveys in the same area. Method refers to how the effective surveyed area was estimated (HMMDM—buffer width of half the mean maximum distance moved of all animals captured in more than one camera-trap station were added to the survey trapping area; Telemetry—home range size estimates based on radio-tracked animals were used to inform the buffer width added to the survey trapping area, SCR—information on capture history of individuals in combination with spatial information of captures were used to directly estimate density).

Country	Study site	Ecoregion	Density	Method	Source
Brazil	Morro do Diabo State Park	Atlantic forest	31.3	HMMDM	[40]
Brazil	Reserve of UNIDERP	Pantanal	56.4	HMMDM	[4]
Brazil	Estância Ecológica SESC Pantanal	Pantanal	11.2	HMMDM	[21]
Bolivia	Ravelo, Kaa-lya del Gran Chaco National Park	Transitional Chaco-Chiquitano dry forest	59*	HMMDM	[6]
Bolivia	San Miguelito, Kaa-lya del Gran Chaco National Park	Transitional Chaco-Chiquitano dry forest	56	HMMDM	[6]
Bolivia	Cerro Cortado, Kaa-lya del Gran Chaco National Park	Chaco dry forest	29.5*	HMMDM	[6]
Bolivia	Tucavaca, Kaa-lya del Gran Chaco National Park	Transitional Chaco-Chiquitano dry forest	29*	HMMDM	[6]
USA, Texas	Yturria Ranch	Western Gulf coastal grassland	30	HMMDM	[41]
Argentina	Urugua-í	Atlantic forest	13.3	HMMDM	[42]
Argentina	Iguazú National Park	Atlantic forest	19.9	HMMDM	[42]
Belize	Chiquibul Forest Reserve and National Park	Broad-leaf rain-forest	25.8	HMMDM	[43]
Belize	Mountain Pine Ridge Forest Reserve	Belizean pine forest	3.1	HMMDM	[43]
Brazil	Ilha do Cardoso State Park	Atlantic forest	40	HMMDM	[44]
Brazil	Feliciano Miguel Abdala Reserve	Atlantic forest	52.1	HMMDM	[45]
Brazil / Argentina	Iguaçú / Iguazú National Parks and San Jorge Forest Reserve	Atlantic forest	16.8	HMMDM	[14]
Argentina	Yabotí Biosphere Reserve	Atlantic forest	8.6	HMMDM	[14]
Brazil	Caraguatá Reserve	Atlantic forest	4	HMMDM	[46]
Brazil	Ponte Branca—ESEC MPL	Atlantic forest	17	HMMDM	[47]
Brazil	Seis R	Atlantic forest	25	HMMDM	[47]
Brazil	Santa Mônica	Atlantic forest	62	HMMDM	[47]
Panama	Darién National Park	Tropical wet forest	62.7	HMMDM	[48]
Peru	Oil concession (Block 39)	Amazon forest	84.8*	HMMDM	[38]
Costa Rica	Talamanca Caribbean Biological Corridor	Tropical wet forest	6.5	Telemetry	[49]
Colombia	Sociedad Civil Palmarito Natural Reserve	Colombian llanos	11	HMMDM	[50]
Brazil	Serra da Capivara National Park	Semi-arid Caatinga	4.5	HMMDM	[5]
Bolivia	Palmar, Kaa-lya del Gran Chaco National Park	Transitional Chaco-Chiquitano dry forest	77	HMMDM	[34]
Bolivia	Estación Isoso, Kaa-lya del Gran Chaco National Park	Chaco-Amazon forest	11*	HMMDM	[34]
Mexico	Sierra Abra-Tanchipa Biosphere Reserve	Tropical deciduous forest	2.9	SCR	[32]
Brazil	Amanã Sustainable Development Reserve	Amazon forest	28.9*	HMMDM	this study
Brazil	Amanã Sustainable Development Reserve	Amazon forest	24.84*	SCR	this study

Our SCR-based estimates were at least 3 times lower than that predicted by the correlation with latitude and rainfall presented by Di Bitetti et al. [14]. We believe that environmental factors acting on a smaller scale than rainfall and latitude, such as habitat conditions or prey availability, can have great influence on ocelot density and distribution. For example, in the seasonally flooded forests of the Mamirauá Reserve, which borders Amanã Reserve and has similar latitude and rainfall values [27], over 10 years of intense camera trapping (overall effort over 15000 camera-trap nights) have yielded only a single ocelot record, suggesting that Mamirauá Reserve holds no resident ocelot population (Ramalho, E. unpublished data). According to Di Bitetti et al. [14], ocelot population density should be relatively high throughout most of the Amazon region, but our results lower that expectation and suggest that density of ocelots throughout the Amazon might be highly variable.

Our study corroborates the notion that surveys designed for larger felids can be used to estimate density of smaller species [25]. Indeed, small felid conservation would benefit if studies designed for larger species could incorporate a nested grid within the main camera-trap array [51,52]. This increases the chance for recaptures at multiple traps, which would improve estimation of movement parameters and baseline encounter rates of species with smaller home ranges. In particular, data obtained from a nested design could be used for small felid density estimation throughout the research area.

When information on population sizes is not available, assessing the conservation status of species has to be based on the extent of their geographic distribution range [53], which can be very uninformative. Status assessments of the ocelot and other small felid species would greatly benefit if their densities were estimated in multiple locations of their distribution using robust analytical methods such as those used in the present study. This is especially true for the vast forests of the Amazon region, which are considered a safeguard for many felid species. Yet there remains a large knowledge gap concerning the population status of most of these species.

## Supporting Information

S1 File Results from the non-spatial capture-recapture models and spatial capture-recapture model with independent estimation of parameters for each survey. (PDF)

S2 File Data file. (PDF)
