# Supplementary material for: Ocelot (Leopardus pardalis) Density in Central Amazonia
Source: PLoS One. 2016 May 18;11(5):e0154624. doi: 10.1371/journal.pone.0154624 (PMC4871438; doi:10.1371/journal.pone.0154624)
Supplement: S1 File — (PDF) [file pone.0154624.s001.pdf]

Ocelot (*Leopardus pardalis*) density in Central Amazonia

Daniel G. Rocha, Rahel Sollmann, Emiliano E. Ramalho, Renata Ilha, Cedric K. W. Tan

S1 File – Results from the non-spatial capture-recapture models and spatial capture-recapture model with independent estimation of parameters for each survey.

Non-spatial capture-recapture models

Methods

We implemented closed non-spatial capture-recapture models in program CAPTURE 2 (Hines, 2010) for each survey separately. We assigned the first operational day of each camera-trap station as the beginning of the first sampling occasion (Soisalo and Cavalcanti, 2006; Trolle and Kéry, 2003). Then we collapsed every consecutive 5-day trapping period into a single trapping occasion to create the individual capture history with 8, 12 and 9 occasions for the first, second and third survey, respectively (Otis et al., 1978). Collapsing of sampling occasions increases probability of detection and may, in turn, increase the precision of population size estimates (Dillon and Kelly, 2007).

In CAPTURE it is possible to provide estimators for models with different assumptions on the variation of detection probability such as, M0 (capture probability constant for all individuals on all sampling occasions), Mh (heterogeneity in capture probability among individuals), Mb (initial capture probability differs from recapture probability), Mt (capture probability varies by occasion), and models that account for combinations of those sources of variation (Mth, Mbh and Mtb) (Burnham and Anderson, 2002; for more details on the models see Otis et al., 1978).

We considered Mh the most biologically plausible amongst the candidate models because the ocelot is a territorial species, resulting in unequal access to the camera trap grid by different individuals (Tobler and Powell, 2012). There is no biological reason to believe that detections of ocelots in the surveyed area vary with time (seasonality effect) or behavior (as bait had no significant effect on photographic rate). Therefore, we reported results from model Mh using the jackknife estimator (Noss et al., 2012).

We divided the population size estimate generated under model Mh by the estimated effective sampled area of the camera-trap survey to estimate density. The effective sampled area is usually calculated by adding a buffer around each camera-trap station (Karanth and Nichols, 1998; Silver et al., 2004), as the area used by captured individuals is certainly larger than the area covered by the trapping grid. In theory, the width of the buffer is related to the home range size of the target species in the study area. In the absence of home range size data, the mean of the maximum distance moved (MMDM) by all individuals photographed at more than one camera-trap station is used as an approximation of the home range diameter. Most studies of ocelot have used the ½ MMDM buffer to estimate effective sampled area (Di Bitetti et al., 2006; Maffei et al., 2005; Trolle and Kéry, 2003). However, some studies have demonstrated that full MMDM buffer may be a better proxy of home range size than ½ MMDM (Dillon and Kelly, 2008; Maffei and Noss, 2008).

We combined individual movements from all surveys to calculate MMDM and shared this value across surveys. As for the spatial capture-recapture analyses, by combining movement information from all surveys we assumed that there is no significant variation on home range sizes across surveys. As recommended

by Dillon and Kelly (2007) for sparse data, we included zero-distance movements (animals captured multiple times but always at the same trap) in the MMDM calculation. For the sake of comparison with previous studies, we reported densities using both buffer widths (full and ½ MMDM) as estimates of effective sampled area. As for the spatial capture-recapture analysis, we used a habitat mask to exclude water surface areas. The standard error for density estimates followed (Karanth and Nichols, 1998).

Results

The mean maximum distance moved by individuals captured more than once (MMDM) was 2919.7 m (range: 0 – 10812 m, SD = 3304.7, N = 15). The effective surveyed area with MMDM buffer was 276.3 km^2 for the first and third survey and 281.3 km^2 for the second survey. We report the summary of population size and density estimates for different effective sampled areas in S1 Table.

S1 Table. *Population size estimates (N) and density estimates (D) with 95% confidence intervals for ocelot in Amanã Reserve generated by CAPTURE under model Mh in three surveys (2013, 2014 and 2015). Density is reported in number of ocelots per 100 km^2 according to two different estimates of effective surveyed area (full and ½ MMDM). SE = standard error; LCI = lower confidence interval; UCI = upper confidence interval.*

Survey	Population size				Density (individuals/100 km^2)							
					MMDM				1/2 MMDM			
	N	SE	LCI	UCI	D	SE	LCI	UCI	D	SE	LCI	UCI
**2013**	33	7.4	25	56	12.1	5.1	1.2	23.0	17.9	8.7	0.0	36.4
**2014**	76	16.1	54	119	27.4	11.3	3.4	51.5	40.1	19.1	0.0	80.8
**2015**	53	13.5	35	90	19.5	8.5	1.3	37.7	28.7	14.0	0.0	59.4

Spatial capture-recapture model with independent estimation of parameters for each survey

Instead of sharing parameters across surveys, we specified a model that independently estimates parameters and density for each survey separately (independent model), using the same procedure described in the Methods section.

The estimates for the independent model had larger confidence interval for two of the three surveys (S1 Fig.). Whereas density estimates for the shared parameters model vary from 20.8 to 25.4 ocelots per 100km^2, estimates for the independent model vary from 10.4 to 73.0 ocelots per km^2 (S2 Table). This higher variability of the density estimates for the independent model is mostly due to differences in the movement parameter estimates across surveys (Tobler and Powell, 2012).

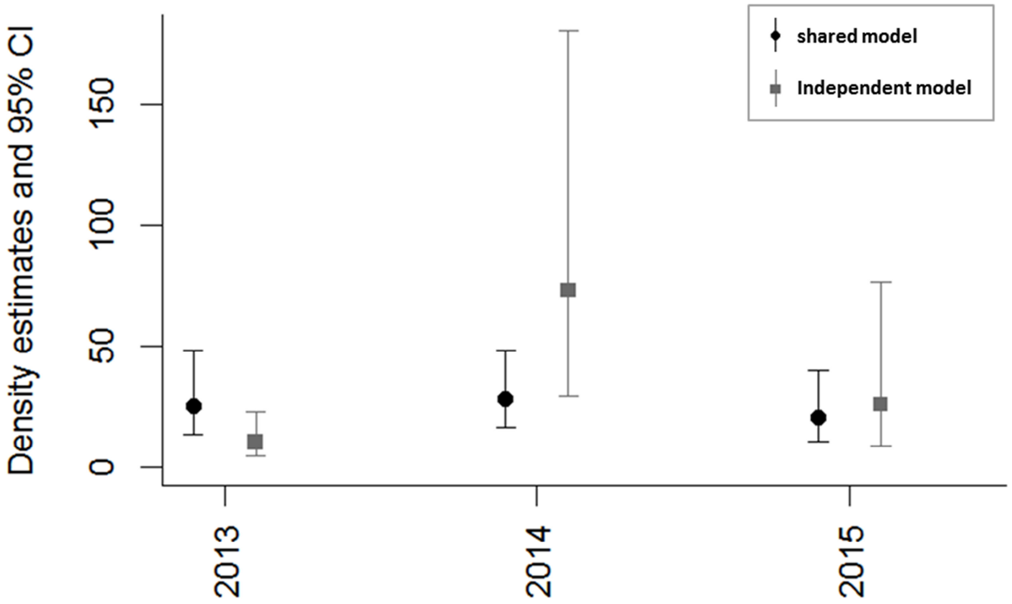

S1 Figure. *Ocelot density estimate and 95% confidence interval for the spatial capture-recapture model fit to camera trapping data from Amanã Reserve. Results from two competing models are reported. For the first model (shared model), data from the three surveys were used to estimate the shared movement parameter σ and encounter rate* λ_0. *For the second model, parameters and density were estimated using only data from each survey separately (independent model). Densities are reported in ocelots per 100 km*^{2}.

***S2 Table**. Ocelot density estimate with standard error (SE) and 95% confidence interval (LCI and UCI) of parameters for spatial capture recapture model fit to camera trapping data from Amanã Reserve. Results from two competing models are reported. For the first model (shared), data from the three surveys were used to estimate the shared movement parameter σ and encounter rate* $\boldsymbol{\lambda_0}$*. For the second time parameters and density were estimated using only data from each survey separately (2013, 2014 and 2015). Densities are reported in ocelots per 100 km*^{2}*.*

	Estimate	SE	LCI	UCI
**Shared**				
σ	2.21	0.33	1.65	2.96
λ_0	0.002	0.001	0.001	0.003
Density 2013	25.5	8.5	13.5	48.1
Density 2014	28.2	8.0	16.4	48.6
Density 2015	20.9	7.1	10.9	39.8
**2013**				
σ	4.45	1.60	2.25	8.80
λ_0	0.001	0.001	0.000	0.004
Density	10.4	4.4	4.7	23.2
**2014**				
σ	0.42	0.08	0.30	0.60
λ_0	0.015	0.007	0.006	0.038
Density	73.0	35.6	29.5	180.4
**2015**				
σ	1.93	0.66	1.01	3.71
λ_0	0.002	0.001	0.000	0.007
Density	25.9	15.5	8.8	76.8

As discussed in the methods section, there is no biological reason to presume that ocelot home range sizes varied across surveys. Therefore, the variation of the movement parameter estimates (ranging from 0.4 to 4.4 km) for the independent model is likely due to the small sample sizes in each survey. For

example, in 2014, only one individual was ever recaptured at more than one trap, rendering estimates of σ for that year non-representative and unreliable. Even though the independent model had lower AICc than the shared parameters model (deltaAICc = 37.1), we report the shared model estimates because parameter estimates are based on a larger sample size and the model is biologically plausible.

Literature cited

Burnham, K.P., Anderson, D.R., 2002. Model selection and multimodel inference: a practical information-theoretic approach. Springer Science & Business Media.

Di Bitetti, M.S., Paviolo, A., De Angelo, C., 2006. Density, habitat use and activity patterns of ocelots (Leopardus pardalis) in the Atlantic Forest of Misiones, Argentina. J. Zool. 270, 153–163. doi:10.1111/j.1469-7998.2006.00102.x

Dillon, A., Kelly, M.J., 2007. Ocelot Leopardus pardalis in Belize: the impact of trap spacing and distance moved on density estimates. Oryx 41, 469–477. doi:10.1017/S0030605307000518

Dillon, A., Kelly, M.J., 2008. Ocelot home range, overlap and density: Comparing radio telemetry with camera trapping. J. Zool. 275, 391–398. doi:10.1111/j.1469-7998.2008.00452.x

Hines, J.E., 2010. Capture 2. Patuxent: USGS- PWRC [WWW Document]. URL

http://www.mbr-pwrc.usgs.gov/software/capture.html.

Karanth, K.U., Nichols, J.D., 1998. Estimation of tiger densities in India using photographic captures and recaptures. Ecology 79, 2852–2862.

Maffei, L., Noss, A.J., 2008. How small is too small? Camera trap survey areas and density estimates for ocelots in the Bolivian Chaco. Biotropica 40, 71–75. doi:10.1111/j.1744-7429.2007.00341.x

Maffei, L., Noss, A.J., Cuéllar, E., Rumiz, D.I., 2005. Ocelot (Felis pardalis) population densities, activity, and ranging behaviour in the dry forests of eastern Bolivia: data from camera trapping. J. Trop. Ecol. 21, 349–353. doi:10.1017/S0266467405002397

Noss, A.J., Gardner, B., Maffei, L., Cuéllar, E., Montaño, R., Romero-Muñoz, A., Sollmann, R., O'Connell, A.F., 2012. Comparison of density estimation methods for mammal populations with camera traps in the Kaa-Iya del Gran Chaco landscape. Anim. Conserv. 15, 527–535. doi:10.1111/j.1469-1795.2012.00545.x

Otis, D.L., Burnham, K.P., White, G.C., Anderson, D.R., 1978. Statistical inference from capture data on closed animal populations. Wildl. Monogr. 3–135.

Silver, S.C., Ostro, L.E.T., Marsh, L.K., Maffei, L., Noss, A.J., Kelly, M.J., Wallace, R.B., Gomez, H., Ayala, G., 2004. The use of camera traps for estimating jaguar Panthera onca abundance and density using capture/recapture analysis. Oryx 38, 148–154. doi:10.1017/S0030605304000286

Soisalo, M.K., Cavalcanti, S.M.C., 2006. Estimating the density of a jaguar population in the Brazilian Pantanal using camera-traps and capture–recapture sampling in combination with GPS radio-telemetry. Biol. Conserv. 129, 487–496. doi:10.1016/j.biocon.2005.11.023

Tobler, M.W., Powell, G.V.N., 2012. Estimating jaguar densities with camera traps: problems with current designs and recommendations for future studies. Biol. Conserv. 159, 109–118. doi:10.1016/j.biocon.2012.12.009

Trolle, M., Kéry, M., 2003. Estimation of ocelot density in the Pantanal using capture–recapture analysis of camera-trapping data. J. Mammal. 84, 607–614.
