# Supplementary material for: Ocelot (Leopardus pardalis) Density in Central Amazonia
Source: PLoS One. 2016 May 18;11(5):e0154624. doi: 10.1371/journal.pone.0154624 (PMC4871438; doi:10.1371/journal.pone.0154624)
Supplement: S2 File — (PDF) [file pone.0154624.s002.pdf]

Ocelot (*Leopardus pardalis*) density in Central Amazonia

Daniel G. Rocha, Rahel Sollmann, Emiliano E. Ramalho, Renata Ilha, Cedric K. W. Tan

S2 File – Data File

```
###==================================================================================================================###
###                                                    DATA FILE                                                      ###
###==================================================================================================================###

### NON-SPATIAL CAPTURE RECAPTURE DATA

## CAPTURE HISTORY FILES (for CAPTURE2)
# 2013
TITLE='Ocelot_Amana_2013_collaps5'
TASK READ CAPTURES X matrix OCCASIONS=8 CAPTURES=8
FORMAT='(8x,a3,1x,8f1.0)'
READ INPUT DATA
RAM  13 THA 10000000
RAM  13 ALI 10000000
RAM  13 GUI 01000001
RAM  13 DIO 01000010
RAM  13 DA5 01000000
RAM  13 DM1 01000000
RAM  13 PED 01000011
RAM  13 DM2 00100001
RAM  13 MOD 00100100
RAM  13 DM3 00010001
RAM  13 GAB 00010000
RAM  13 RAC 00010000
RAM  13 ARI 00010000
RAM  13 FLA 00001000
RAM  13 BOB 00000100
RAM  13 DF1 00000100
RAM  13 LAI 00000100
RAM  13 TAY 00000010
RAM  13 MAR 00000010
TASK POPULATION ESTIMATE JACKKN MH-CHA

# 2014
TITLE='Ocelot Amana 2014_collaps5'
TASK READ CAPTURES X matrix OCCASIONS=12 CAPTURES=12
FORMAT='(8x,a3,1x,12f1.0)'
READ INPUT DATA
```

```
RAM  14 GAB 100000000000
RAM  14 ALI 100000000000
RAM  14 LOU 100100000000
RAM  14 THY 010000000001
RAM  14 DIO 010000000000
RAM  14 SET 010000100000
RAM  14 DA1 010000010000
RAM  14 ANA 010000000000
RAM  14 PAT 010000000000
RAM  14 TAN 001000000000
RAM  14 THA 001000000000
RAM  14 RAF 001000000000
RAM  14 KEL 001000000000
RAM  14 LEO 000100000000
RAM  14 DA4 000100000000
RAM  14 SAM 000100000000
RAM  14 MAR 000100000000
RAM  14 NAT 000010000000
RAM  14 LIB 000001000000
RAM  14 VII 000001001000
RAM  14 BIR 000001000000
RAM  14 TAY 000001011100
RAM  14 JUA 000001000000
RAM  14 KAM 000001000000
RAM  14 ROD 000001000000
RAM  14 LAB 000000100000
RAM  14 PED 000000010000
RAM  14 DEB 000000010000
RAM  14 RAS 000000010000
RAM  14 DA3 000000010000
TASK POPULATION ESTIMATE JACKKN MH-CHA

# 2015
TITLE='Ocelot Amana 2015_collaps5'
TASK READ CAPTURES X matrix OCCASIONS=9 CAPTURES=9
FORMAT='(8x,a3,1x,9f1.0)'
READ INPUT DATA
RAM  15 RF1 100000000
RAM  15 DIO 100000000
RAM  15 RM2 100000000
RAM  15 RM9 100000000
RAM  15 M10 001000000
RAM  15 RM1 001000100
RAM  15 PED 000110000
RAM  15 RF2 000100000
RAM  15 RM7 000010000
```

RAM 15 RF5 000010000
RAM 15 RF6 000001000
RAM 15 RM5 000000100
RAM 15 RF3 000000100
RAM 15 THA 000000100
RAM 15 RM8 000000010
RAM 15 BIR 000000010
RAM 15 RF4 000000001
TASK POPULATION ESTIMATE JACKKN MH-CHA

SPATIAL CAPTURE-RECAPTURE DATA

CAPTURE HISTORY FILE

3013-2014-2105

#Session	ID	Occasion	Detector	Sex
2013	THA	5	23	m
2013	ALI	7	17	f
2013	GUI	12	5	m
2013	FLA	28	9	m
2013	TAY	37	17	f
2013	GUI	39	1	m
2013	DIO	46	29	m
2013	A5	49	46	NA
2013	M1	50	41	m
2013	PED	50	41	m
2013	M2	51	43	m
2013	M3	54	31	m
2013	MOD	56	41	m
2013	RAC	58	40	f
2013	GAB	58	45	f
2013	ARI	61	47	f
2013	BOB	66	32	m
2013	MOD	67	40	m
2013	LAI	67	16	f
2013	F1	68	40	f
2013	DIO	73	38	m
2013	MAR	73	43	f
2013	PED	74	41	m
2013	PED	78	41	m
2013	M2	80	37	m
2013	M3	81	35	m
2014	A1	10	17	NA
2014	A1	36	17	NA
2014	A3	92	20	NA

2014	A4	19	26	NA
2014	ALI	33	111	f
2014	ANA	60	31	f
2014	BIR	84	46	m
2014	DEB	39	12	f
2014	DIO	65	110	m
2014	GAB	57	40	f
2014	JUA	80	20	f
2014	KAM	87	109	f
2014	KEL	68	42	f
2014	LAB	89	114	m
2014	LEO	20	7	m
2014	LIB	78	32	f
2014	LOU	62	46	f
2014	LOU	75	45	f
2014	MAR	72	36	f
2014	NAT	76	28	f
2014	PAT	14	6	m
2014	PED	89	37	m
2014	RAF	14	9	m
2014	RAS	43	33	m
2014	ROD	34	13	m
2014	SAM	19	5	m
2014	SET	62	41	f
2014	SET	85	41	f
2014	TAN	68	35	f
2014	THA	13	18	m
2014	TAY	28	17	f
2014	TAY	40	17	f
2014	TAY	43	17	f
2014	TAY	48	17	f
2014	TAY	49	17	f
2014	THY	64	111	m
2014	THY	116	111	m
2014	VII	78	32	f
2014	VII	95	32	f
2015	RF1	4	2	f
2015	RF2	18	4	f
2015	RF3	34	11	f
2015	RF4	89	29	f
2015	RF5	71	32	f
2015	RF6	30	33	f
2015	RM1	33	2	m
2015	RM1	59	29	m
2015	RM10	53	41	m
2015	BIR	79	50	m

2015	RM2	5	8	m
2015	DIO	89	16	m
2015	DIO	44	27	m
2015	THA	39	18	m
2015	RM5	34	21	m
2015	PED	68	28	m
2015	PED	60	43	m
2015	RM7	68	29	m
2015	RM8	80	30	m
2015	RM9	46	38	m

TRAP LAYOUT (EPSG32720)

2013

#Detector	X	Y	String	Covariates
1	315394.91	9730340.44	00011111111111111111111111111111111111110000000000000000000000000000000000000000000	/Baited
2	318492.61	9722052.04	00111111111111111111111111111111111111000000000000000000000000000000000000000000000	/Baited
3	318153.76	9726243.61	00001111111111111111111111111111111111110000000000000000000000000000000000000000000	/Baited
4	316696.56	9721126.45	11111111111111111111111111111111111111100000000000000000000000000000000000000000000	/Baited
5	321291.62	9721334.55	00000111111111111111111111111111111111111000000000000000000000000000000000000000000	/Baited
6	313778.79	9725956.17	01111111111111111111111111111111111111000000000000000000000000000000000000000000000	/Baited
7	313053.38	9727914.73	01111111111111111111111111111111111111000000000000000000000000000000000000000000000	/Baited
8	316660.41	9725108.33	00111111111111111111111111111111111111000000000000000000000000000000000000000000000	/Baited
9	318889.93	9724510.66	00000111111111111111111111111111111111111000000000000000000000000000000000000000000	/Baited
10	319684.29	9720695.64	00111111111111111111111111111111111111000000000000000000000000000000000000000000000	/Baited
11	315033.12	9724364.30	11111111111111111111111111111111111111100000000000000000000000000000000000000000000	/Baited
12	320406.77	9724625.32	00000111111111111111111111111111111111111000000000000000000000000000000000000000000	/Baited
13	313699.71	9729584.19	01111111111111111111111111111111111111000000000000000000000000000000000000000000000	/Baited
14	318273.07	9729624.10	00001111111111111111111111111111111111110000000000000000000000000000000000000000000	/Baited
15	317418.75	9723449.50	00000111111111111111111111111111111111000000000000000000000000000000000000000000000	/Baited
16	319830.79	9726342.97	00000000000000000000000000000000000000011111111111111111111111111111111111111100000	/Baited
17	316708.78	9729151.13	00001111111111111111111111111111111111110000000000000000000000000000000000000000000	/Baited
18	315110.36	9728400.53	00011111111111111111111111111111111111000000000000000000000000000000000000000000000	/Baited
19	320299.95	9722861.49	00000111111111111111111111111111111111111000000000000000000000000000000000000000000	/Baited
20	322046.60	9723273.87	00000000000000000000000000000000000000011111111111111111111111111111111111111100000	/Baited
21	319149.37	9727886.90	00000011111111111111111111111111111111110000000000000000000000000000000000000000000	/Baited
22	315545.92	9722535.98	11111111111111111111111111111111111111100000000000000000000000000000000000000000000	/Baited
23	315657.47	9726689.46	00011111111111111111111111111111111111000000000000000000000000000000000000000000000	/Baited
24	317939.53	9719676.14	11111111111111111111111111111111111111100000000000000000000000000000000000000000000	/Baited
25	317089.87	9727670.96	00001111111111111111111111111111111111110000000000000000000000000000000000000000000	/Baited
26	316955.66	9730992.56	00000111111111111111111111111111111111110000000000000000000000000000000000000000000	/Baited
27	323485.38	9725382.07	00000000000000000000000000000000000000000011111111111111111111111111111111111111111	/Baited
28	320304.21	9730320.98	00000000000000000000000000000000000000111111111111111111111111111111111111111110000	/Baited
29	321458.17	9725192.75	00000000000000000000000000000000000000011111111111111111111111111111111111111100000	/Baited
30	320932.31	9728709.54	00000000000000000000000000000000000000111111111111111111111111111111111111111110000	/Baited
31	321328.20	9727074.56	00000000000000000000000000000000000000111111111111111111111111111111111111111110000	/Baited

32	323615.77	9721482.26	00000000000000000000000000000000000000011111111111111111111111111111111111111100000	/Baited
33	311964.17	9729076.66	01111111111111111111111111111111111111000000000000000000000000000000000000000000000	/Baited
34	320772.36	9732258.84	00000000000000000000000000000000000000001111111111111111111111111111111111111111100	/Baited
35	326876.66	9725610.26	00000000000000000000000000000000000000000111111111111111111111111111111111111111100	/Baited
36	323139.92	9727818.73	00000000000000000000000000000000000000001111111111111111111111111111111111111111100	/Baited
37	322783.05	9729577.56	00000000000000000000000000000000000000001111111111111111111111111111111111111111100	/Baited
38	325787.26	9721855.30	00000000000000000000000000000000000000000111111111111111111111111111111111111110000	/Baited
39	325689.40	9723711.72	00000000000000000000000000000000000000000111111111111111111111111111111111111100000	/Baited
40	325437.04	9725503.82	00000000000000000000000000000000000000000111111111111111111111111111111111111111110	/Baited
41	323333.61	9732379.07	00000000000000000000000000000000000000000111111111111111111111111111111111111111110	/Baited
42	324135.04	9730875.09	00000000000000000000000000000000000000000111111111111111111111111111111111111111110	/Baited
43	322238.13	9731405.82	00000000000000000000000000000000000000001111111111111111111111111111111111111111110	/Baited
44	324571.00	9728985.88	00000000000000000000000000000000000000000111111111111111111111111111111111111111000	/Baited
45	326716.91	9727560.89	00000000000000000000000000000000000000000011111111111111111111111111111111111111000	/Baited
46	326352.21	9729388.25	00000000000000000000000000000000000000000011111111111111111111111111111111111111000	/Baited
47	324971.24	9727214.96	00000000000000000000000000000000000000000111111111111111111111111111111111111111000	/Baited
48	323604.48	9723451.58	00000000000000000000000000000000000000011111111111111111111111111111111111111111110	/Baited
49	319021.24	9731749.20	00000000000000000000000000000000000000000011111111111111111111111111111111111111110	/Baited
50	326205.47	9730288.14	00000000000000000000000000000000000000000011111111111111111111111111111111111111110	/Baited

2014

#Detector	X	Y	String	Covariates
1	315394.91	9730340.44	011111111111111111111111111111111111111111111111111100000000000000000000000000000000000000000000000000000000000000000000	/Baited
2	318492.61	9722052.04	000111111111111111111111111111111111111111111111111111000000000000000000000000000000000000000000000000000000000000000000	/Baited
3	318153.76	9726243.61	111111111111111111111111111111111111111111111111111000000000000000000000000000000000000000000000000000000000000000000000	/Baited
4	316696.56	9721126.45	000111111111111111111111111111111111111111111111111111100000000000000000000000000000000000000000000000000000000000000000	/Baited
5	321291.62	9721334.55	111111111111111111111111111111111111111111111111111100000000000000000000000000000000000000000000000000000000000000000000	/Baited
6	313778.79	9725956.17	000011111111111111111111111111111111111111111111111111110000000000000000000000000000000000000000000000000000000000000000	/Baited
7	313053.38	9727914.73	000011111111111111111111111111111111111111111111111111110000000000000000000000000000000000000000000000000000000000000000	/Baited
8	316660.41	9725108.33	000111111111111111111111111111111111111111111111111111000000000000000000000000000000000000000000000000000000000000000000	/Baited
9	318889.93	9724510.66	111111111111111111111111111111111111111111111111111100000000000000000000000000000000000000000000000000000000000000000000	/Baited
10	319684.29	9720695.64	000111111111111111111111111111111111111111111111111111000000000000000000000000000000000000000000000000000000000000000000	/Baited
11	315033.12	9724364.30	000111111111111111111111111111111111111111111111111111100000000000000000000000000000000000000000000000000000000000000000	/Baited
12	320406.77	9724625.32	111111111111111111111111111111111111111111111111111100000000000000000000000000000000000000000000000000000000000000000000	/Baited
13	313699.71	9729584.19	000011111111111111111111111111111111111111111111110000000000000000000000000000000000000000000000000000000000000000000000	/Baited
14	318273.07	9729624.10	111111111111111111111111111111111111111111111111111000000000000000000000000000000000000000000000000000000000000000000000	/Baited
15	317418.75	9723449.50	000111111111111111111111111111111111111111111111111111000000000000000000000000000000000000000000000000000000000000000000	/Baited
16	319830.79	9726342.97	000000000000000000000000000000000000000000000000000111111111111111111111111111111111111111111111111000000000000000000000	/Baited
17	316708.78	9729151.13	111111111111111111111111111111111111111111111111111000000000000000000000000000000000000000000000000000000000000000000000	/Baited
18	315110.36	9728400.53	011111111111111111111111111111111111111111111111111100000000000000000000000000000000000000000000000000000000000000000000	/Baited
19	320299.95	9722861.49	111111111111111111111111111111111111111111111111111100000000000000000000000000000000000000000000000000000000000000000000	/Baited
20	322046.60	9723273.87	000000000000000000000000000000000000000000000000000011111111111111111111111111111111111111111111000000000000000000000000	/Baited
21	319149.37	9727886.90	000011111111111111111111111111111111111111111111111100000000000000000000000000000000000000000000000000000000000000000000	/Baited
22	315545.92	9722535.98	000111111111111111111111111111111111111111111111111111100000000000000000000000000000000000000000000000000000000000000000	/Baited
23	315657.47	9726689.46	011111111111111111111111111111111111111111111111111110000000000000000000000000000000000000000000000000000000000000000000	/Baited
24	317939.53	9719676.14	000111111111111111111111111111111111111111111111111111100000000000000000000000000000000000000000000000000000000000000000	/Baited

25	317089.87	9727670.96	1111111111111111111111111111111111111111111111111110000000000000000000000000000000000000000000000000000000000000000000000	/Baited
26	316955.66	9730992.56	0111111111111111111111111111111111111111111111111110000000000000000000000000000000000000000000000000000000000000000000000	/Baited
27	323485.38	9725382.07	0000000000000000000000000000000000000000000000000000000011111111111111111111111111111111111111111111000000000000000000000	/Baited
28	320304.21	9730320.98	0000000000000000000000000000000000000000000000000001111111111111111111111111111111111111111111111000000000000000000000000	/Baited
29	321458.17	9725192.75	0000000000000000000000000000000000000000000000000000111111111111111111111111111111111111111111111000000000000000000000000	/Baited
30	320932.31	9728709.54	0000000000000000000000000000000000000000000000000001111111111111111111111111111111111111111111110000000000000000000000000	/Baited
31	321328.20	9727074.56	0000000000000000000000000000000000000000000000000001111111111111111111111111111111111111111111110000000000000000000000000	/Baited
32	323615.77	9721482.26	0000000000000000000000000000000000000000000000000000111111111111111111111111111111111111111111111000000000000000000000000	/Baited
33	311964.17	9729076.66	0000111111111111111111111111111111111111111111111111111000000000000000000000000000000000000000000000000000000000000000000	/Baited
34	320772.36	9732258.84	0000000000000000000000000000000000000000000000000000011111111111111111111111111111111111111111111110000000000000000000000	/Baited
35	326876.66	9725610.26	0000000000000000000000000000000000000000000000000000000001111111111111111111111111111111111111111111100000000000000000000	/Baited
36	323139.92	9727818.73	0000000000000000000000000000000000000000000000000000011111111111111111111111111111111111111111111110000000000000000000000	/Baited
37	322783.05	9729577.56	0000000000000000000000000000000000000000000000000000011111111111111111111111111111111111111111111110000000000000000000000	/Baited
38	325787.26	9721855.30	0000000000000000000000000000000000000000000000000000000011111111111111111111111111111111111111111111000000000000000000000	/Baited
39	325689.40	9723711.72	0000000000000000000000000000000000000000000000000000000011111111111111111111111111111111111111111111000000000000000000000	/Baited
40	325437.04	9725503.82	0000000000000000000000000000000000000000000000000000000011111111111111111111111111111111111111111111000000000000000000000	/Baited
41	323333.61	9732379.07	0000000000000000000000000000000000000000000000000000001111111111111111111111111111111111111111111110000000000000000000000	/Baited
42	324135.04	9730875.09	0000000000000000000000000000000000000000000000000000001111111111111111111111111111111111111111111110000000000000000000000	/Baited
43	322238.13	9731405.82	0000000000000000000000000000000000000000000000000000011111111111111111111111111111111111111111111110000000000000000000000	/Baited
44	324571.00	9728985.88	0000000000000000000000000000000000000000000000000000001111111111111111111111111111111111111111111111000000000000000000000	/Baited
45	326716.91	9727560.89	0000000000000000000000000000000000000000000000000000000001111111111111111111111111111111111111111111000000000000000000000	/Baited
46	326352.21	9729388.25	0000000000000000000000000000000000000000000000000000000001111111111111111111111111111111111111111111000000000000000000000	/Baited
47	324971.24	9727214.96	0000000000000000000000000000000000000000000000000000001111111111111111111111111111111111111111111110000000000000000000000	/Baited
48	323604.48	9723451.58	0000000000000000000000000000000000000000000000000000111111111111111111111111111111111111111111111000000000000000000000000	/Baited
49	319021.24	9731749.20	0000000000000000000000000000000000000000000000000001111111111111111111111111111111111111111111110000000000000000000000000	/Baited
50	326205.47	9730288.14	0000000000000000000000000000000000000000000000000000000001111111111111111111111111111111111111111111100000000000000000000	/Baited
101	318352.07	9727228.65	0000000000000000000000000000011111111111111111111111111000000000000000000000000000000000000000000000000000000000000000000	/Unbaited
102	316420.41	9730128.17	0000000000000000000000000000000111111111111111111111111100000000000000000000000000000000000000000000000000000000000000000	/Unbaited
103	316080.29	9727791.46	0000000000000000000000000000000000000000000000000000000011111111111111111111111111111111111111111111000000000000000000000	/Unbaited
104	312459.98	9729973.76	0000000000000000000000000000000000000000000000000000000000111111111111111111111111111111111111111111111111111111111111110	/Unbaited
105	319377.62	9719670.98	0000000000000000000000000000000000000000000000000000000000111111111111111111111111111111111111111111111111111111111111111	/Unbaited
106	318923.73	9723078.40	0000000000000000000000000000000000000000000000000000000000111111111111111111111111111111111111111111111111111111111111110	/Unbaited
107	317063.28	9722229.16	0000000000000000000000000000000000000000000000000000000000111111111111111111111111111111111111111111111111111111111111110	/Unbaited
108	324957.71	9722893.37	0000000000000000000000000000000000000000000000000000000000111111111111111111111111111111111111111111111111111111111111110	/Unbaited
109	322237.29	9728278.75	0000000000000000000000000000000000000000000000000000000000111111111111111111111111111111111111111111111111111111111111111	/Unbaited
110	322389.36	9726216.70	0000000000000000000000000000011111111111111111111111111000000000000000000000000000000000000000000000000000000000000000000	/Unbaited
111	322449.54	9720989.88	0000000000000000000000000000011111111111111111111111111000000000000000000000000000000000000000000000000000000000000000000	/Unbaited
112	320302.63	9729414.47	0000000000000000000000000000001111111111111111111111111100000000000000000000000000000000000000000000000000000000000000000	/Unbaited
113	323743.41	9729956.97	0000000000000000000000000000000111111111111111111111111100000000000000000000000000000000000000000000000000000000000000000	/Unbaited
114	325491.51	9728254.19	0000000000000000000000000000000111111111111111111111111100000000000000000000000000000000000000000000000000000000000000000	/Unbaited

2015

#Detector	X	Y	String	Covariates
1	315394.9089	9730340.44	00000111111111111111111111111111111111111110000000000000000000000000000000000000000000000000	/Unbaited
2	318492.61	9722052.04	01111111111111111111111111111111111111100000000000000000000000000000000000000000000000000000	/Unbaited
3	318153.76	9726243.61	00001111111111111111111111111111111111111100000000000000000000000000000000000000000000000000	/Unbaited

4	316696.5558	9721126.452	1111111111111111111111111111111111111110000000000000000000000000000000000000000000000000000	/Unbaited
5	321291.624	9721334.553	0011111111111111111111111111111111111111100000000000000000000000000000000000000000000000000	/Unbaited
6	313778.787	9725956.166	0000111111111111111111111111111111111111111000000000000000000000000000000000000000000000000	/Unbaited
7	313053.3826	9727914.726	0000111111111111111111111111111111111111111000000000000000000000000000000000000000000000000	/Unbaited
8	316660.4078	9725108.329	0111111111111111111111111111111111111111000000000000000000000000000000000000000000000000000	/Unbaited
9	318889.9293	9724510.662	0011111111111111111111111111111111111111100000000000000000000000000000000000000000000000000	/Unbaited
10	319684.2875	9720695.638	0111111111111111111111111111111111111111000000000000000000000000000000000000000000000000000	/Unbaited
11	315033.119	9724364.303	1111111111111111111111111111111111111110000000000000000000000000000000000000000000000000000	/Unbaited
12	320403.4297	9724625.313	0011111111111111111111111111111111111111100000000000000000000000000000000000000000000000000	/Unbaited
13	313699.7114	9729584.186	0000111111111111111111111111111111111111111000000000000000000000000000000000000000000000000	/Unbaited
14	318260.8647	9729600.867	0001111111111111111111111111111111111111110000000000000000000000000000000000000000000000000	/Unbaited
15	317418.7531	9723449.5	0111111111111111111111111111111111111111000000000000000000000000000000000000000000000000000	/Unbaited
16	319830.7909	9726342.972	0000000000000000000000000000000000000000000111111111111111111111111111111111111111111111110	/Unbaited
17	316708.7826	9729151.128	0001111111111111111111111111111111111111110000000000000000000000000000000000000000000000000	/Unbaited
18	315110.3612	9728400.534	0000011111111111111111111111111111111111111100000000000000000000000000000000000000000000000	/Unbaited
19	320299.9448	9722861.492	0011111111111111111111111111111111111111100000000000000000000000000000000000000000000000000	/Unbaited
20	322046.6028	9723273.873	0000000000000000000000000000000000000000000001111111111111111111111111111111111111111111111	/Unbaited
21	319149.3684	9727886.898	0001111111111111111111111111111111111111110000000000000000000000000000000000000000000000000	/Unbaited
22	315545.9177	9722535.976	1111111111111111111111111111111111111110000000000000000000000000000000000000000000000000000	/Unbaited
23	315657.4704	9726689.455	0000011111111111111111111111111111111111111100000000000000000000000000000000000000000000000	/Unbaited
24	317939.5325	9719676.136	0111111111111111111111111111111111111111000000000000000000000000000000000000000000000000000	/Unbaited
25	317090.9774	9727670.958	0000111111111111111111111111111111111111111000000000000000000000000000000000000000000000000	/Unbaited
26	316955.6619	9730992.555	0000011111111111111111111111111111111111111100000000000000000000000000000000000000000000000	/Unbaited
27	323485.3799	9725382.072	0000000000000000000000000000000000000000011111111111111111111111111111111111111111111110000	/Unbaited
28	320304.2107	9730320.981	0000000000000000000000000000000000000000000011111111111111111111111111111111111111111111110	/Unbaited
29	321458.1676	9725192.75	0000000000000000000000000000000000000000000001111111111111111111111111111111111111111111111	/Unbaited
30	320932.3087	9728709.535	0000000000000000000000000000000000000000000111111111111111111111111111111111111111111111110	/Unbaited
31	321328.2	9727074.56	0000000000000000000000000000000000000000000111111111111111111111111111111111111111111111110	/Unbaited
32	323615.7654	9721482.255	0000000000000000000000000000000000000000000000111111111111111111111111111111111111111111111	/Unbaited
33	311964.1663	9729076.657	0000111111111111111111111111111111111111111000000000000000000000000000000000000000000000000	/Unbaited
34	320772.3559	9732258.836	0000000000000000000000000000000000000000000011111111111111111111111111111111111111111111100	/Unbaited
35	326876.66	9725610.26	0000000000000000000000000000000000000001111111111111111111111111111111111111111111111100000	/Unbaited
36	323139.9211	9727818.73	0000000000000000000000000000000000000000000011111111111111111111111111111111111111111111100	/Unbaited
37	322783.0463	9729577.555	0000000000000000000000000000000000000000000011111111111111111111111111111111111111111111100	/Unbaited
38	325787.2594	9721855.304	0000000000000000000000000000000000000000011111111111111111111111111111111111111111111110000	/Unbaited
39	325689.3959	9723711.721	0000000000000000000000000000000000000000011111111111111111111111111111111111111111111110000	/Unbaited
40	325437.0347	9725503.823	0000000000000000000000000000000000000000011111111111111111111111111111111111111111111110000	/Unbaited
41	323333.6105	9732379.068	0000000000000000000000000000000000000000001111111111111111111111111111111111111111111111000	/Unbaited
42	324135.0353	9730875.087	0000000000000000000000000000000000000000001111111111111111111111111111111111111111111111000	/Unbaited
43	322238.1337	9731405.824	0000000000000000000000000000000000000000000011111111111111111111111111111111111111111111100	/Unbaited
44	324571.0024	9728985.883	0000000000000000000000000000000000000000001111111111111111111111111111111111111111111111000	/Unbaited
45	326716.9049	9727560.894	0000000000000000000000000000000000000001111111111111111111111111111111111111111111111100000	/Unbaited
46	326352.2117	9729388.246	0000000000000000000000000000000000000001111111111111111111111111111111111111111111111100000	/Unbaited
47	324971.2445	9727214.957	0000000000000000000000000000000000000000001111111111111111111111111111111111111111111111000	/Unbaited
48	323604.4798	9723451.579	0000000000000000000000000000000000000000000000111111111111111111111111111111111111111111111	/Unbaited
49	319021.2373	9731749.195	0000000000000000000000000000000000000000000011111111111111111111111111111111111111111111110	/Unbaited

50	326205.4722	9730288.144	000000000000000000000000000000000000000011111111111111111111111111111111111111111111111100000	/Unbaited
